# Recent advances in Major Histocompatibility Complex (MHC) class I antigen presentation: Plastic MHC molecules and TAPBPR-mediated quality control

**DOI:** 10.12688/f1000research.10474.1

**Published:** 2017-02-17

**Authors:** Andy van Hateren, Alistair Bailey, Tim Elliott

**Affiliations:** 1 Institute for Life Sciences and Cancer Sciences Unit, University of Southampton, Southampton, UK

**Keywords:** major histocompatibility complex, class I antigen presentation, peptide loading, plasticity

## Abstract

We have known since the late 1980s that the function of classical major histocompatibility complex (MHC) class I molecules is to bind peptides and display them at the cell surface to cytotoxic T cells. Recognition by these sentinels of the immune system can lead to the destruction of the presenting cell, thus protecting the host from pathogens and cancer. Classical MHC class I molecules (MHC I hereafter) are co-dominantly expressed, polygenic, and exceptionally polymorphic and have significant sequence diversity. Thus, in most species, there are many different MHC I allotypes expressed, each with different peptide-binding specificity, which can have a dramatic effect on disease outcome.

Although MHC allotypes vary in their primary sequence, they share common tertiary and quaternary structures. Here, we review the evidence that, despite this commonality, polymorphic amino acid differences between allotypes alter the ability of MHC I molecules to change shape (that is, their conformational plasticity). We discuss how the peptide loading co-factor tapasin might modify this plasticity to augment peptide loading. Lastly, we consider recent findings concerning the functions of the non-classical MHC I molecule HLA-E as well as the tapasin-related protein TAPBPR (transporter associated with antigen presentation binding protein-related), which has been shown to act as a second quality-control stage in MHC I antigen presentation.

## Loading of MHC I with peptides is a two-step iterative process

**Figure 1.**
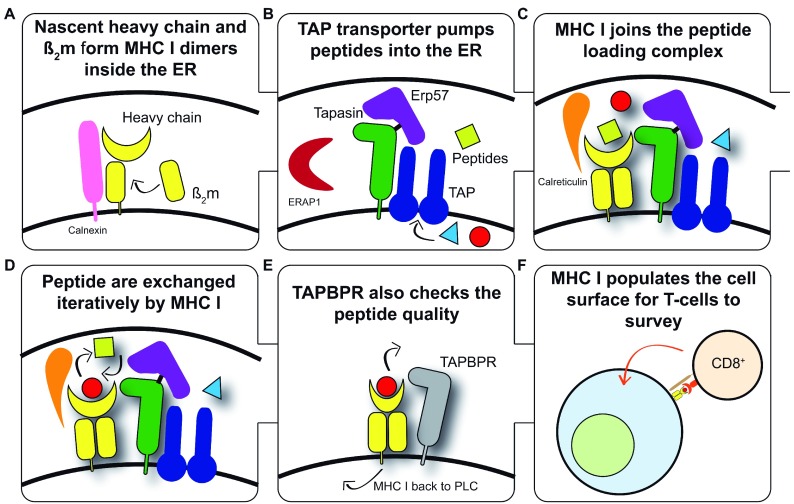
An overview of major histocompatibility complex (MHC) I antigen processing and presentation. ( **A**) Nascent MHC I heavy chains (HCs) fold in the endoplasmic reticulum (ER). The co-ordinated activities of the ER-resident enzymes dolichyl-diphosphooligosaccharide protein glycotransferase and glucosidases I and II generate the mono-glucosylated N-linked glycan required for HCs to interact with the chaperone calnexin, which together with ERp57 monitor the glycosylation and oxidative status of HCs and facilitate the formation of non-covalently bound HC-beta _2_ microglobulin (HC-β _2_m) heterodimers. ( **B**) A proportion of the intracellular proteome is pumped into the ER via the transporter associated with antigen presentation (TAP). TAP supplies peptides for the consideration of MHC I molecules for binding. Each TAP heterodimer associates with up to two molecules of tapasin, each of which is disulphide-linked to ERp57. Aminopeptidases are also present within the ER and can trim peptides to their optimal length for MHC I binding. ( **C**) MHC I becomes loaded with peptides while associated with the peptide-loading complex (PLC). MHC–β _2_m heterodimers are escorted to the PLC by calreticulin ^81^, where the weak interactions that exist between individual components of the PLC are synergistically strengthened as part of the PLC. ( **D**) MHC I undergoes tapasin-mediated peptide exchange. All MHC I allotypes have an intrinsic ability to optimise their peptide cargo which is enhanced by the action of tapasin and the PLC. Once a sufficiently stable peptide–MHC I complex is formed, dissociation from the PLC occurs. ( **E**) TAP binding protein-related (TAPBPR)-mediated quality control of the MHC I peptide repertoire. TAPBPR is not a member of the PLC and appears to function further along the secretory pathway. TAPBPR refines the peptide repertoire by the removal of low-affinity peptides from MHC I molecules. ( **F**) Peptide–MHC I complexes present a proportion of the intracellular proteome to the immune system. Most species express several MHC I allotypes (six in humans), each of which is capable of binding a variety of peptides. The expression of multiple copies of each MHC I allotype at the cell surface cumulatively allows the internal health to be efficiently monitored by cytotoxic T cells.

In order to present a snapshot of the intracellular protein content, nascent MHC I heavy chains (HCs) non-covalently associate with β _2_-microglobulin (β _2_m) ( Figure 1A) and form a tri-molecular complex with a peptide. Binding of β _2_m and peptide to HC is co-operative and stabilises the trimer ^1, 2^. The peptides presented by MHC I molecules are predominantly generated in the cytoplasm and are transported into the endoplasmic reticulum (ER) by the transporter associated with antigen presentation (TAP) ( Figure 1B), where they are considered for binding to MHC I molecules. Loading of MHC I with peptides occurs while MHC I–β _2_m heterodimers are incorporated into the peptide-loading complex (PLC) ( Figure 1C) comprising one TAP heterodimer, two molecules of tapasin each covalently bound to a molecule of ERp57 and non-covalently bound to an MHC I molecule, and two calreticulin molecules per complex ^3, 4^.

Most of the peptides pumped into the ER by TAP will bind to whichever MHC I allotypes are present, but very few are likely to complement the specificity-determining pockets of the MHC I allotype completely. Therefore, most peptides transported into the ER are unlikely to bind with high enough affinity to stabilise the MHC I molecules sufficiently to allow effective antigen presentation ^5, 6^. Therefore, efficient peptide selection cannot ensue if MHC I molecules simply bind the first peptide that they encounter in the PLC. Evidence suggests that peptide loading is an iterative process in which an initial, suboptimal peptide cargo is bound followed by rounds of peptide exchange gradually replacing this with a cargo of a high average affinity, producing stable MHC I molecules and conferring increased expression at the cell surface ( Figure 1D) ^7, 8^. Investigations into this peptide exchange by Garstka *et al.* showed that all peptides bind to MHC I molecules at similar rates but that suboptimal peptides dissociate more rapidly at physiological temperatures, which is discussed in detail later ^9^.

The efficiency of peptide optimisation varies between MHC I allotypes. This is most apparent when the loading co-factor tapasin is not expressed ^10^ or is unable to function because of viral immune-evasion molecules ^11– 13^ and the intrinsic “peptide selector” function of MHC I allotypes is revealed. Allotypes with poor peptide selector function depend upon tapasin to optimise their peptide repertoire ^8, 14^. Point mutations in either MHC I or tapasin that prevent MHC I from binding to the PLC limit the ability of “tapasin-dependent” MHC I allotypes to select a high-affinity cargo but do not prevent peptide binding ^7, 15– 18^. Until recently, it has not been clear why even a single amino acid polymorphism between allotypes could change the manner in which MHC I molecules assemble with peptides. Before we consider how tapasin augments peptide loading, we will discuss the mechanistic basis by which “tapasin-independent” classical MHC I allotypes select and assemble with high-affinity peptides without assistance from tapasin and the PLC.

## MHC I allotypes are plastic and differ in their ability to explore different conformations

Comparison of the numerous peptide–MHC I X-ray crystallographic structures that are available shows that although they share a common fold, they are not super-imposable structures; subtle differences are apparent ^19^. It is clear from these structures, and a recent study ^9^, that the peptide-binding domain can undergo quite marked structural rearrangements in order to accommodate peptides, some of which may bind suboptimally because they are longer or have incompatible residues for the pockets of the MHC I allotype. Supporting these crystallographic observations, early experiments showed that peptide binding to MHC I can alter recognition by antibodies specific for particular conformations of MHC I molecules (for example, ^20– 23^) or result in conformation-specific changes in the intramolecular transfer of fluorescence (for example, ^24^). Analysis of peptide–MHC I interactions by fluorescence energy transfer or fluorescence anisotropy experiments also supports the notions that peptide, β _2_m, and HC binding are synergistic and that peptide binding or dissociation involves a change in the conformation of MHC I molecules ^1, 25^.

The conformation of peptide–MHC I complexes appears to be influenced not just by the MHC I molecule but also by the peptide that is bound, which can have profound effects on T-cell recognition (for example, differential recognition of two peptides presented by HLA-A*02:01 by the A6 T-cell receptor ^26^). Protein crystallography has shown that although the two unligated peptide–MHC I complexes closely resemble each other, there are differences in peptide, T-cell receptor, and the A*02:01 molecule itself when the two T-cell-ligated peptide–MHC I structures are compared. A combination of fluorescence anisotropy, molecular dynamics simulations (MDS), and crystallography experiments attributed the different interfaces formed with the A6 T-cell receptor to variations in the molecular motions of the peptide, which in turn resulted in differences of the motions of the A*02:01 molecule. Hawse *et al.* extended this study by comparing complexes of HLA-A*02:01 loaded with these or other peptides via hydrogen-deuterium exchange (HDX) and mass spectrometry as well as fluorescent anisotropy experiments ^27^. The results allowed the authors to infer peptide-dependent flexibility of the HLA-A*02:01 peptide-binding domain, which included the α helices and β-sheet floor. HDX reports on motions on the millisecond timescale and slower, MDS reports on motions up to the microsecond timescale, whilst fluorescence anisotropy reports motions on the nanosecond timescale motions. These findings, collected using a range of experimental techniques and sampling different timescales, demonstrate that peptide–MHC I complexes are not static structures but are intrinsically conformationally flexible, plastic molecules.

The importance of plasticity for determining the function of proteins has been extensively demonstrated. For example, a variety of ways have been described in which signals are passed within and between cells, reviewed in ^28^, where the initiation of a signalling event modulates the conformation of an upstream signalling molecule, which in turn alters the dynamic properties of downstream components of the signalling pathways in order to propagate the signal. This has prompted investigations to determine whether differences in plasticity might explain the variable intrinsic ability of MHC I allotypes to select and assemble with high-affinity peptides.

Characterisation of the conformational status of MHC I molecules prior to peptide loading has been complicated by the propensity of empty MHC I molecules to aggregate and denature. However, nuclear magnetic resonance (NMR) spectral and biophysical analyses suggest that the peptide-binding domain undergoes significant conformational alteration in the absence of bound peptide ^29, 30^. In contrast, however, the three-dimensional structures of the α3 domain and β _2_m remain relatively insensitive to the presence or absence of peptide. NMR spectroscopy is the best technique for observing protein dynamics and can report on conformational changes over a range of timescales, from picoseconds to milliseconds and slower ^31^. Yanaka *et al.* used NMR to show that while the membrane-proximal α3 domains of HLA-B*35:01 molecules loaded with a panel of peptides were similar, the peptide-binding domains exhibited peptide-dependent conformational fluctuations over a wide area ^32^. These findings are supported by those of Sgourakis *et al.*, who also observed several flexible regions of a truncated peptide–MHC I complex by NMR ^33^. Yanaka *et al.* also found that the peptide-binding domain of peptide–MHC I complexes transiently exists in two conformational states, which they designated as major and minor states. The proportions of molecules adopting each state varied according to the bound peptide; however, for each of the peptide–MHC I complexes studied, the minor state formed tighter contacts with the peptide than did the major state. This led the authors to propose that peptide binding occurs via a two-step transient induced-fit model, in which a peptide is loosely bound by the major state and then conformational rearrangements occur as the MHC I molecule transitions to the minor state in which the peptide is bound more tightly. These findings mirror observations made with MHC class II molecules, which present peptides to helper T cells expressing CD4 co-receptors and which suggest that alternative MHC class II conformations are required for peptide exchange to occur ^34^.

Collectively, these observations imply that the primary sequence not only changes the chemistry of the peptide-binding site but also determines the intrinsic plasticity of the peptide-binding site. As discussed below, the emerging principle appears to be that more conformational plasticity will allow MHC molecules to visit multiple different conformations, which in turn is likely to increase the ability to bind and exchange peptides. Attempts are now being made to provide a full mechanistic description of MHC peptide selector function in terms of MHC protein dynamics.

## A plasticity-based mechanism for peptide optimisation

Several groups have employed MDS to investigate the peptide-empty state of MHC I, which confirms that MHC I exhibits more conformational plasticity in the absence of peptide, consistent with NMR and biophysical data ^23, 35– 40^. Reassuringly, MDS localises much of the molecular motions to regions of the peptide-binding domain implicated in both crystallographic ^9^ and HDX ^27^ experiments of peptide–MHC complexes.

**Figure 2.**
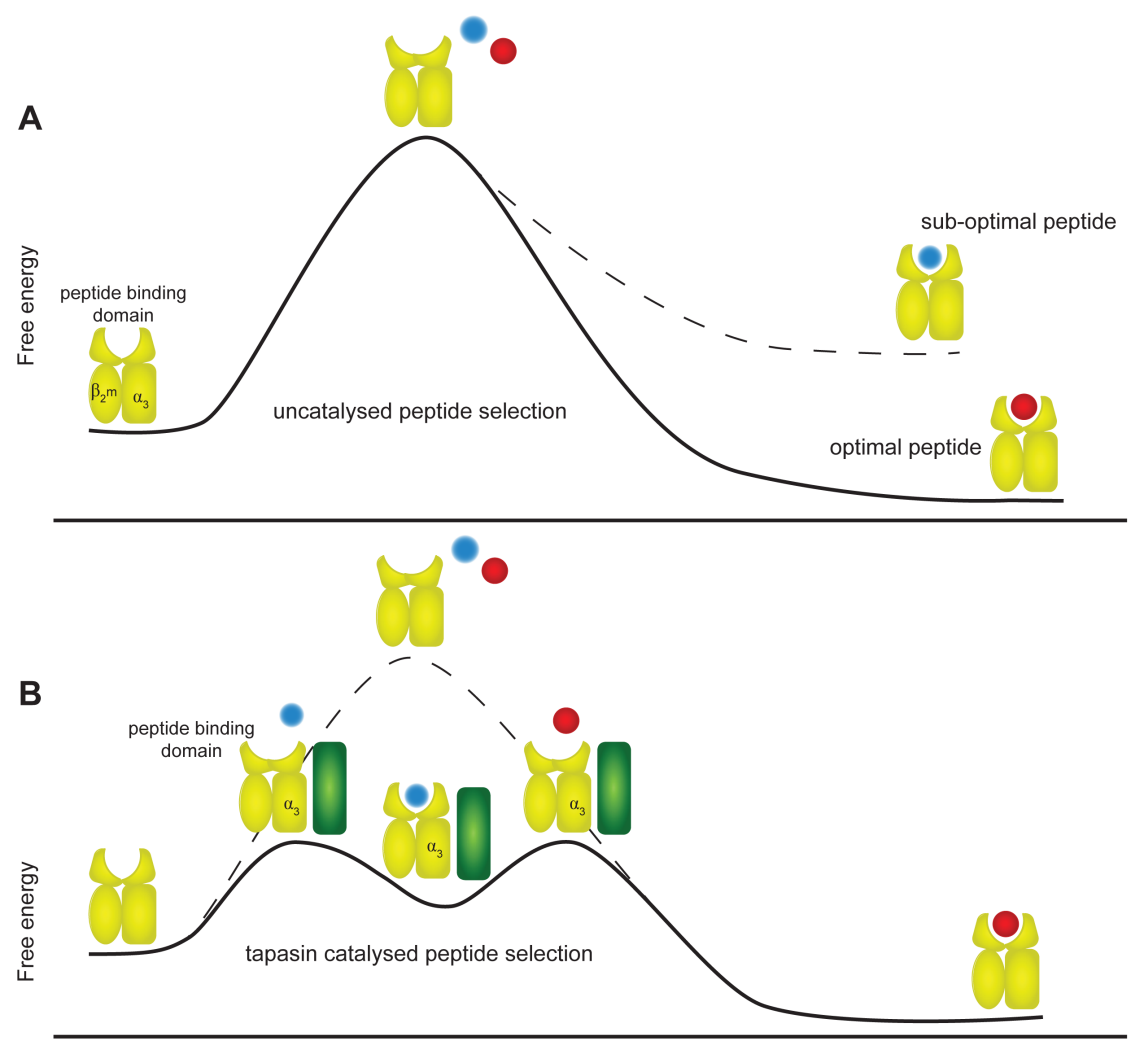
Plasticity in major histocompatibility complex (MHC) I peptide selection. ( **A**) In an uncatalysed reaction, the intrinsic ability of MHC I (yellow) to select peptide is determined by the plasticity encoded into each allotype by its primary sequence. Some allotypes are intrinsically more able to sample the higher-energy peptide-receptive conformations than others and therefore have better intrinsic peptide selector function. The degree of complementarity between the peptide and MHC I allotype in stabilising the peptide-receptive conformation then determines whether the peptide is selected leading to a stable native conformation or whether the iterative peptide exchange process continues. ( **B**) In a tapasin-catalysed reaction, tapasin (green) modulates MHC I allotypes to first enhance sampling of the peptide-receptive conformations and then destabilises peptide binding to enhance exchange of suboptimal peptides for optimal peptides. The modulation of MHC conformation by tapasin occurs via interactions at both the peptide-binding domain and the membrane-proximal α3 domain, leading to faster peptide exchange, which in turn leads to increased presentation of MHC I molecules at the cell surface.

Comparison of different MHC I allotypes by MDS reveals different intrinsic levels of plasticity ^37, 39– 42^. We have observed a correlation where MHC I allotypes possessing an intrinsic ability to select high-affinity peptides are better able to explore distinct conformational states compared with allotypes with poor selector function ^39, 40, 43^. We have hypothesised that the rate that different intermediate structures are sampled dictates how often conformations conducive to peptide binding are adopted. Increased adoption of peptide-binding conformations in turn will facilitate peptide exchange, culminating in the selection of a high-affinity peptide and the closed, compact MHC I conformation ( Figure 2A).

Fitting a range of kinetic models to experimental data, we found that the model that most closely predicted experimental observations is one which involves a conformational change occurring in MHC I during peptide selection, as suggested by NMR and biophysical analyses ^1, 2, 25, 39, 44^. The model makes two mechanistic predictions: firstly, that the intrinsic differential peptide selector function of MHC I allotypes correlates with the rate at which MHC I transits from an open, peptide-receptive conformation to the closed (crystallisable) conformation and, secondly, that the rate that peptide-loaded complexes revert from closed to open conformations depends upon the affinity with which the peptide is bound. Taken together, this suggests that MHC I allotypes with fast “closing” rates will get to the peptide-dependent re-opening stage faster. Thus, in an iterative process, these allotypes will optimise their peptide cargo more efficiently as a result of the faster and greater progression through these cycles ^45– 48^.

## Allosteric communication within MHC I

Unsurprisingly, most reports of MHC I plasticity are focused on the dynamics of the peptide-binding domain, as demonstrated by the NMR, MDS, HDX, and fluorescence anisotropy experiments discussed above. However, MDS analysis of the dynamic motions of the entire peptide–MHC I complex reveals significant flexibility between the peptide binding and the membrane-proximal α3 and β _2_m domains ( Figure 2A) ^39, 40, 43^. In the absence of peptide, MDS analysis showed that those MHC I allotypes with good intrinsic peptide selector function sampled distinct conformational populations, defined by the orientations between the membrane-proximal α3 domain and peptide-binding domains. These coupled “domain-domain” motions were not observed for allotypes with poor peptide selector function ^39, 40, 43^.

Allostery in proteins, which can be defined as a change in protein structure and function at one site resulting from the modification of a distant site, has long been considered an essential part of protein function, modulating protein plasticity and sending signals in a variety of ways ^28, 49^. For example, at one extreme, cyclic AMP (cAMP) binding to the cAMP-binding domain of the catabolite activator protein (CAP, also known as cAMP receptor protein) causes the reorientation and activation of the CAP DNA-binding domain without large conformational rearrangements occurring ^50^. At the other extreme, oxygenation of haemoglobin initiates large domain rearrangements ^51^. It is therefore conceivable that the binding of peptides to MHC I might modulate a conserved allosteric network ^52^ and lead to population shifts amongst the ensemble of MHC I conformations ^34^.

The observation that efficient peptide selector function correlates with the co-ordinated positioning of the α3 and β _2_m domains relative to the peptide-binding domain suggests that allosteric domain-domain communication is a key determinant of MHC I peptide selection properties. This suggests that movements of the membrane-proximal domains must be aligned with those of the peptide-binding domain in order for peptide-binding or dissociation events to occur and are not restricted exclusively to the dynamic events involving just the peptide-binding domain.

The notion that the dynamic properties of the α3 and β _2_m domains are linked to those of the peptide-binding domain raises the possibility that receptors interacting with the membrane-proximal domains, which are invariant in sequence in most species, may interact differently depending on the allosteric influence of the peptide-binding domain dynamics. One obvious candidate is the CD8 co-receptor ^53^. Additionally, there are several examples where the inhibitory receptors on natural killer (NK) cells bind to the membrane-proximal MHC I domains (reviewed in 54). One interesting case is the murine homodimeric NK Ly49 receptor family that bind to a cavity beneath the peptide-binding domain of MHC I molecules comprising residues of the α2, α3, and β _2_m domains. Ly49 receptors have been shown to adopt open or closed conformations, which determines whether the dimer interacts bivalently or monovalently with MHC I molecules. Furthermore, most Ly49 receptors can participate in either *cis* or *trans* interactions with MHC I (that is, MHC I molecules that are expressed on the same or different cell surfaces).

## Plasticity as the mechanism by which tapasin enhances MHC I peptide selection

Since the identification of tapasin in the mid-1990s, the multiple ways in which tapasin enhances peptide selection have been well documented: tapasin clusters peptide-receptive MHC I molecules around the TAP peptide transporter within the PLC ^55– 59^; tapasin enhances the quantity of MHC I molecules and the rate at which they are presented; and tapasin improves discrimination between peptides, ensuring that MHC I molecules are loaded with high-affinity peptides ^8, 60, 61^. The extent to which tapasin assists peptide loading varies between MHC I allotypes ^10^, but in doing so tapasin normalises the differences in intrinsic peptide selection between allotypes such that in the presence of tapasin there is equally rapid and efficient peptide selection by all allotypes ^8, 14^.

Moreover, *in vitro* experiments have shown that tapasin achieves its function by accelerating peptide binding and dissociation rates ^62, 63^, and direct binding experiments showed that tapasin preferentially binds peptide-empty MHC I molecules and that peptide binding enhances the dissociation of peptide–MHC I complexes from tapasin ^47, 63^.

These observations suggest that tapasin binds to peptide-empty MHC class I molecules and enhances maintenance of a peptide-receptive state from which peptides bind and dissociate rapidly. Following peptide binding, the closed (native) MHC I state is adopted, from which peptides dissociate more slowly and from which tapasin dissociates. Such a possibility is consistent with the notion that tapasin catalyses both the closure of open peptide–MHC I conformations and the peptide-dependent re-opening of closed peptide–MHC I conformations ^39, 47, 48^.

Two sites of interaction have been identified between tapasin and MHC I via targeted mutagenesis studies and molecular docking simulations ^16, 48, 64, 65^. Understandably, much attention has focussed on the interaction involving the N-terminal domain of tapasin and the MHC I peptide-binding domain. Recently, it has been suggested that this interaction is centred on two loops (residues 12–18 and 77–85) of tapasin binding the MHC I α _2-1_ helix, with the underlying β _7,8_ strands of MHC I binding to a loop in tapasin (residues 187–196) ^48^. Indeed, a mechanism by which tapasin enhances peptide selection was proposed by the authors involving the N-terminal domain of tapasin pulling on the β-sheet floor of the MHC I peptide-binding domain to widen the peptide-binding domain, opposing the peptide-induced forces attempting to close the peptide-binding domain ^48^.

However, the interaction between the membrane-proximal domain of tapasin and the MHC I α3 domain (involving residues 299, 328, 330, 333, 334, 335, and 345 of tapasin interacting with MHC I residues 222–227 and 229 ^48^) is also important for tapasin-assisted MHC I selector function as indicated by numerous mutagenesis studies ^17, 18, 62^. The functional relevance of this membrane-proximal interaction is supported by two further findings: firstly, that mutagenesis of a natural polymorphism in the α3 domain of chicken MHC I allotypes, that is predicted to participate in this interaction, influences the ability of tapasin to enhance peptide dissociation ^63^; secondly, that mimicking the membrane-proximal interaction between the α3 domain and tapasin increases the exploration of different peptide-binding domain conformations ^39^.

Taken together, these observations are consistent with a model where tapasin binding to MHC I via two distinct sites modulates the plasticity of the bound MHC I molecule in such a way as to shift the equilibrium towards peptide-receptive MHC I conformations ( Figure 2B). In such a state (or states), peptides are sampled by MHC I more rapidly, leading to enhanced selection of high-affinity peptides. In this speculative model, interaction between tapasin and the membrane-proximal α3 domain of MHC I is important for two reasons: firstly, it may have an allosteric effect on the dynamic properties of the peptide-binding domain modulating plasticity such that conformations that can bind peptides rapidly are more likely to be adopted. Secondly, simultaneous engagement of the α3 and peptide-binding domain sites by tapasin might align these domains in such a way as to preferentially stabilise an orientation that is optimal for peptide exchange. This conformation would predispose to rapid peptide-binding kinetics and rapid transition to the closed conformation ^39, 40, 43^.

In summary, similar to the picture emerging for MHC class II molecules and their peptide loading co-factor molecule named DM ^34^, we hypothesise that the plasticity of MHC I allotypes is modulated by tapasin to allow greater exploration of conformations that catalyse and enhance peptide exchange. Therefore, the rate of selection of high-affinity peptides is increased via a mechanism that is underpinned by modulation of protein plasticity ^39^.

## The non-classical molecule HLA-E

In comparison with classical MHC I molecules, non-classical MHC I molecules (including HLA-E, -F, and -G in humans) are non-polymorphic or have limited polymorphism, have a restricted expression pattern, and are recognised by specific T-cell receptor subsets or by innate immune receptors. Recently, vaccine-encoded peptides presented by HLA-E were shown to confer protection against simian immunodeficiency virus in macaques via CD8 T-cell responses ^66– 69^.

Normally, HLA-E molecules bind peptides derived from the signal sequences of classical MHC I molecules and present these to NK cells bearing CD94/NKG2 receptors ^70^. However, it has become apparent that HLA-E molecules can bind a wider selection of peptides than canonical MHC I leader sequence-derived peptides and that these peptides can be presented to CD8 T cells ^68, 71– 73^. Interestingly, many of these “atypical” peptides do not match the canonical HLA-E peptide motif, suggesting that the different types of peptides are bound in different ways. Comparison by MDS of HLA-E to HLA-A*02:01, which share similar peptide-binding preferences, revealed that HLA-E has a more rigid peptide-binding groove, which significantly remains open even in the absence of peptide, in contrast to classical MHC I molecules, such as HLA-A*02:01, whose empty grooves collapse inwards ^68^. Docking simulations suggest that the atypical peptide repertoire can be accommodated by binding higher in the HLA-E groove, where there is a different chemical environment from that experienced by the deeply anchored canonical leader sequence-derived peptides.

Whether the leader sequence peptides are loaded into HLA-E molecules and subsequently exchanged for different peptides or whether different HLA-E binding groove chemistries are exposed to the peptidome depending on the “ground-state” conformation of HLA-E remains to be seen. However, it seems likely that conformational plasticity of the rigid peptide-binding groove of HLA-E, perhaps even co-ordinated with the α3 domain, will play a role.

## TAPBPR

The gene encoding TAPBPR was identified in 2002 ^74^, but it has only recently been shown that TAPBPR is involved in MHC I peptide loading ^75– 78^. Despite having only 22% homology with tapasin at the sequence level ^74^, TAPBPR is predicted to fold in a similar way to tapasin and uses an equivalent site of interaction to bind to MHC I:β _2_m molecules as tapasin does ^16, 75, 76^. Like tapasin, TAPBPR preferentially binds peptide-empty MHC I and is displaced by peptide binding to MHC I, increases the rate that peptides bind to peptide-receptive MHC, enhances dissociation of peptides from peptide–MHC I complexes, and increases discrimination between peptides ^77, 78^. These findings are consistent with the observation that TAPBPR influences the rate that MHC I molecules transit through the secretory pathway and the incorporation of MHC I molecules into the PLC ^75^.

Despite these similarities, functional differences show that TAPBPR is not a simple duplicate of tapasin. For example, while tapasin deficiency severely downregulates the expression of most MHC I allotypes ^79, 80^, TAPBPR deficiency has only a modest effect ^75^. Also, while tapasin is the keystone in the PLC, TAPBPR does not interact with ERp57 or any other PLC members and may function in secretory compartments downstream of the ER/cis Golgi ^75^. Furthermore, differences are apparent in the functional properties of tapasin and TAPBPR as measured by fluorescence polarisation assays, where dissociation of some peptides was enhanced by one protein more so than the other, or where binding or dissociation of certain peptides was unaffected by TAPBPR but augmented by tapasin ^77^. Further compelling data attesting to the function of TAPBPR in MHC I antigen presentation are that TAPBPR deficiency or overexpression alters the number and the nature of the peptides presented by MHC I allotypes ^77^. Analysis of the MHC I immunopeptidome, cell surface expression levels, and thermal stability of MHC I molecules expressed in TAPBPR-deficient or TAPBPR-overexpressing cells suggests that TAPBPR functions to further focus the repertoire of peptides presented at the cell surface after tapasin-mediated loading and editing in the PLC ^77^.

This difference in the function of the two MHC I-specific co-factors may relate to the environment in which the two proteins operate: tapasin in a compartment rich in competing peptides delivered to the PLC, and TAPBPR in an environment where peptides are likely to be less abundant. Here, peptide dissociation rather than peptide exchange may be the prevailing TAPBPR-catalysed function.

## Concluding remarks

There is a huge array of potential peptide epitopes that could bind to MHC I molecules and be presented to the immune system, but only a small fraction are actually bound by MHC I molecules and presented. Given that cytotoxic T cells can bring about the destruction of the presenting cell, understanding the processes governing peptide selection is of fundamental importance. Any attempts we might one day wish to make to manipulate the immune response will surely be more likely to succeed if they are based on a deep mechanistic understanding of how particular peptides are selected for presentation in the presence or absence of tapasin and edited further by TAPBPR.

Perhaps the biggest recent advance concerning MHC I antigen presentation is that we have greater appreciation that MHC I molecules, particularly in the empty state, are dynamic proteins that bend, flex, twist, and crunch with consummate ease. The conformational plasticity of MHC I molecules carries functional significance in that the ability of the empty MHC I molecules to adopt different conformations determines how MHC I allotypes assemble with peptides. We suggest that a key component determining the conformational plasticity of the entire MHC I molecule is the coupling of motions between membrane-proximal and peptide-binding domains. These ideas may also extend to non-classical MHC I molecules such as HLA-E.

We have proposed that the loading co-factor tapasin uses its dual interaction surfaces to modulate the conformational plasticity of MHC I molecules and increase the exploration of different MHC I conformations. This allows MHC I molecules to sample peptide-receptive conformations more frequently and enhances selection and assembly with high-affinity peptides.

Lastly, it has recently become clear that TAPBPR fine-tunes the MHC I peptide repertoire. Whereas *in vitro* experiments have generally shown that tapasin and TAPBPR possess many similar functional properties, results from cellular studies generally show that these MHC I-specific co-factors have opposing functions. Given the similarities in structure, interaction surfaces and *in vitro* peptide-binding assays, we suggest that the opposing function of these proteins is not related to differences in the ability of these proteins to modulate MHC I plasticity but instead is dependent on the subcellular environment in which the proteins operate.

## Abbreviations

β _2_m, beta _2_ microglobulin; cAMP, cyclic AMP; CAP, catabolite activator protein; ER, endoplasmic reticulum; HC, heavy chain; HDX, hydrogen deuterium exchange; HLA, human leukocyte antigen; MDS, molecular dynamics simulation; MHC, major histocompatibility complex; NK, natural killer; NMR, nuclear magnetic resonance; PLC, peptide-loading complex; TAP, transporter associated with antigen presentation; TAPBPR, TAP binding protein-related.
